# Implementation and 8-year follow-up of an uninterrupted oxygen supply system in a hospital in The Gambia

**DOI:** 10.5588/ijtld.15.0889

**Published:** 2016-08-01

**Authors:** B. D. Bradley, J. D. Light, A. O. Ebonyi, P. C. N'Jai, R. C. Ideh, B. E. Ebruke, E. Nyassi, D. Peel, S. R. C. Howie

**Affiliations:** * Centre for Global Engineering and Department of Chemical Engineering and Applied Chemistry, University of Toronto, Toronto; † Engineering Science, University of Toronto, Toronto, Ontario, Canada; ‡ Medical Research Council Unit The Gambia, Fajara, The Gambia; § Ashdown Consultants, Hartfield, UK; ¶ Department of Paediatrics, University of Auckland, Auckland; # Centre for International Health, University of Otago, Dunedin, New Zealand

**Keywords:** oxygen concentrators, oxygen cylinders, The Gambia, Africa, cost analysis

## Abstract

SETTING: A 42-bed hospital operated by the Medical Research Council (MRC) Unit in The Gambia.

OBJECTIVE: To devise, test and evaluate a cost-efficient uninterrupted oxygen system in the MRC Hospital.

DESIGN: Oxygen cylinders were replaced with oxygen concentrators as the primary source of oxygen. An uninterruptable power supply (UPS) ensured continuity of power. Hospital staff were trained on the use of the new system. Eight years post-installation, an analysis of concentrator maintenance needs and costs was conducted and user feedback obtained to assess the success of the system.

RESULTS: The new system saved at least 51% of oxygen supply costs compared to cylinders, with savings likely to have been far greater due to cylinder leakages. Users indicated that the system is easier to use and more reliable, although technical support and staff training are still needed.

CONCLUSION: Oxygen concentrators offer long-term cost savings and an improved user experience compared to cylinders; however, some technical support and maintenance are needed to upkeep the system. A UPS dedicated to oxygen concentrators is an appropriate solution for settings where power interruptions are frequent but short in duration. This approach can be a model for health systems in settings with similar infrastructure.

## RESUME

CONTEXTE : Un hôpital de 42 lits géré par une unitédu Medical Research Council (MRC) en Gambie.

OBJECTIF : Elaborer, tester etévaluer un système de fourniture d'oxygène ininterrompu rentable pour l'hôpital MRC.

SCHÉMA : Les cylindres d'oxygène ont été remplacés par des concentrateurs d'oxygène comme première source d'oxygène. Un système de fourniture de courant ininterrompu (UPS) a résolu le problème de continuité du courant. Le personnel de l'hôpital a été formé à l'utilisation de ce nouveau système. Huit années après son installation, une analyse des besoins et des coûts de maintenance du concentrateur a été réalisée et le feedback des usagers a été obtenu afin d'évaluer le succès du système.

RÉSULTATS : Le nouveau système a épargné au moins 51% des coûts de fourniture d'oxygène par comparaison aux cylindres, avec des économies susceptibles d'avoir été bien plus importantes en raison de fuites des cylindres. Les usagers ont déclaré que le système était plus facile à utiliser et plus fiable, bien qu'un soutien technique et une formation du personnel soient encore nécessaires.

CONCLUSION : Les concentrateurs d'oxygène permettent des économies à long terme et l'expérience des usagers est meilleure que celle des cylindres; cependant, le système requiert un certain soutien technique et une maintenance pour fonctionner au mieux. Une UPS dédiée aux concentrateurs d'oxygène est une solution appropriée dans les lieux qui souffrent de coupures de courant fréquentes mais de courte durée. Cette approche peut être un modèle pour les systèmes de santé dans des contextes d'infrastructures similaires.

## RESUMEN

MARCO DE REFERENCIA: Un hospital de 42 camas administrado por la Unidad del Consejo de Investigaciones Médicas (MRC) en Gambia.

OBJETIVO: Idear, poner a prueba y evaluar un sistema ininterrumpido y rentable de administración de oxígeno en el hospital del MRC.

MÉTODO: Los cilindros de oxígeno se remplazaron por concentradores de oxígeno como fuentes primarias de abastecimiento. La continuidad del suministro eléctrico se obtuvo mediante un sistema de alimentación ininterrumpida (UPS). Se capacitó al personal del hospital sobre el uso del nuevo sistema. Ocho años después de la instalación, se llevó a cabo un análisis de las necesidades de mantenimiento del concentrador y sus costos y se obtuvo retroalimentación por parte de los usuarios con el fin de evaluar la eficacia del sistema.

RESULTADOS: El nuevo sistema ahorró como mínimo un 51% del suministro de oxígeno, en comparación con el uso de los cilindros; es posible que el ahorro haya sido mucho mayor, habida cuenta de las fugas en los cilindros. Los usuarios afirmaron que el sistema es más sencillo y más fiable, aunque se precisa aún una mejor asistencia técnica y más capacitación del personal.

CONCLUSIÓN: Los concentradores de oxígeno ofrecen ahorros de costos a largo plazo y mejoran la experiencia de los usuarios, en comparación con el uso de cilindros; sin embargo, es necesario aportar asistencia técnica y capacitación con el fin de mejorar el mantenimiento del sistema. Un UPS dedicado a los concentradores de oxígeno es una solución apropiada en los entornos donde son frecuentes las interrupciones del suministro eléctrico de corta duración. Esta estrategia puede constituir un modelo para los sistemas de salud en otros entornos con infraestructuras semejantes.

OXYGEN IS AN ESSENTIAL MEDICINE for the treatment of severe childhood illnesses such as pneumonia, malaria and sepsis, as well as obstetric emergencies.^1^ Many health facilities in developing countries struggle to provide this life-saving treatment.^2^,^3^ Oxygen cylinders, the traditional supply method, are expensive and logistically awkward, while oxygen concentrators, although typically more cost-efficient,^4^,^5^ require reliable power, which is absent in many low-resource countries^2^,^6–8^ such as The Gambia. Located in West Africa, The Gambia has a population of 1.8 million, a gross national income (GNI) per capita of US$1620 and an annual under-five mortality of 74 per 1000.^9^

The Medical Research Council (MRC) Unit in The Gambia operates a 42-bed hospital near the capital, Banjul, admitting around 1000 patients annually. The hospital is well-staffed and caters for both children and adults. The case mix, which comprises infectious and non-infectious diseases, is primarily determined by patients self-referring from the community. There are usually several patients at any one time on oxygen (five on average), pulse oximeters are available, and standard guidelines are used for oxygen therapy. The Unit supports a Biomedical Engineering (BME) Department that manages and maintains the clinical and laboratory equipment at the Unit.^10^ The site has electricity from the national grid and back-up generators, but suffers power interruptions several times a week, typically of a few minutes up to half an hour (rarely). For example, during a monitored period from November 2011 to September 2012, on average about 15 interruptions per week were recorded. Mean interruption duration was about 1 min, with a maximum recorded interruption of 38 min (data from the MRC Facilities Department).

The MRC Hospital, which relied on cylinder oxygen, sought a more cost-efficient uninterrupted oxygen solution for both its own services and as a potential model for other Gambian health facilities. This paper describes the process of identifying and scoping the oxygen supply problem in The Gambia, devising and testing a solution in the MRC Hospital, evaluating that solution and drawing lessons that could be applied elsewhere in The Gambia and similar settings.

## METHODS

### Identifying and scoping the oxygen problem

In 2003, one of the authors (SH) observed problems with the oxygen supply in hospitals in The Gambia. In 2004, a team consisting of members from the Gambian Government, the World Health Organization and the MRC Unit conducted a situational analysis of oxygen availability and treatment in The Gambia.^7^ Only three of 12 health facilities assessed had a reliable oxygen supply. Poor electricity and maintenance capacity hampered the use of concentrators, and logistical and cost limitations meant that supplies from cylinders were unreliable. While the majority of the facilities (58%) had power for less than 12 h per day, two facilities had 12–19 h, and three had nearly 24 h of power per day.^7^ General user perceptions of oxygen equipment were that cylinders were simple to operate but expensive, awkward to move and prone to running out without warning. Facilities with piped oxygen (MRC Hospital and one other) were losing most of their cylinder supply through leakage, adding to the expense. Concentrators were more portable, but needed reliable electricity and were susceptible to breakdown.^7^

Given these distinct advantages and disadvantages, a further analysis was conducted to compare these options taking into account costs, reliability, usability and logistics.^11^ Based on a short-term (86 days) prospective study at the MRC's 42-bed hospital, directly measured cost savings of using concentrators were well in excess of 50%.^11^ These data provided the impetus for devising and testing a long-term concentrator-based system at the MRC Hospital.

### Devising and testing an improved oxygen system at the MRC Hospital

Starting in 2004, a new system was phased in that consisted of oxygen concentrators as the main source of oxygen, cylinders as a back-up source and an uninterruptable power supply (UPS) system (8.0 kVA rated APC Symmetra LX, Schneider Electric, West Kingston, RI, USA) to deal with power interruptions. The wards were fitted with new wiring and electrical outlets so that the concentrators could be plugged directly into the UPS supply. In the event of a power outage, continuity of supply to these outlets is instantaneous and automatic. The capacity of the UPS installed can support one 590W concentrator for 100 min or five concentrators (mix of 350W and 590W) for about 23 min. An added benefit of the UPS is that it provides consistency of voltage when mains or generator supply is of poor quality.

Four concentrators were initially purchased, eight were added in 2007 and a final two were added in 2009 and 2011. All concentrators were Intensity (590W), Elite (350W), or Visionaire (290W) models by Airsep (Buffalo, NY, USA); the first two models have independent data to support their use in tropical settings.^12^,^13^ An initial stock of spare parts was purchased with the concentrators, and additional parts were ordered from the manufacturer as needed. Neither the UPS nor the concentrators were under service contracts with suppliers; all maintenance was done in-house. Routine preventive maintenance for the concentrators was performed by the BME Department. All PM jobs and repairs were documented in an electronic equipment management database. The Facilities Department was responsible for maintenance of the UPS. Hospital staff were trained in the use of the system, and training was incorporated into the general induction for new staff members.

### Evaluating the oxygen system performance and costs

In 2012, a team consisting of the Matron and a paediatrician from the MRC Hospital, the MRC's Child Survival research theme head, the MRC's senior Biomedical Engineering Technologist (BMET) and two engineers from Canada was formed to assess the oxygen system. In July 2012, the team sought feedback from users (eight nurses and two physicians) in a focus group format as to their experiences before and after the introduction of the new system, an approach used in the hospital as part of its routine service development and clinical audit activities. Members of the focus group had worked in the hospital prior to 2004 when cylinders were the primary source of oxygen. The focus was on the themes of reliability and usability. Members of the assessment team documented the comments anonymously, and then filled in an assessment questionnaire based on team consensus.

Data from the BME Department dating back to when the concentrators were introduced up until August 2013 (published in a parallel study^14^), provided a retrospective account of the performance and maintenance costs of the 14 concentrators at the MRC Hospital. This informed a comparison of the costs of the new system against the cost to deliver a comparable amount of oxygen via cylinders. Detailed data on UPS maintenance were not available; however, the UPS has not yet needed a complete replacement and neither the power module nor the battery module (key components needing replacement in the event of unit malfunction) were replaced for the first 2 years. A separate cost estimate for UPS maintenance is included in the results.

The activities reported here were undertaken as part of an operational project approved by the Gambian Government-Medical Research Council Joint Ethics Committee, Banjul, The Gambia (SCC/EC974).

## RESULTS

### User experience

Users felt that the previous oxygen supply from cylinders was relatively reliable. Staff members were not always sure if the cylinders were full on delivery, and it was reported that the adapters attaching the cylinders to the piping were prone to leakage. Users reported that the reliability of supply with the new concentrator-based system was very good, better than with the cylinders, and that oxygen has always been available when needed. The longest power outage recalled by users was about 20 min, and the UPS held out for that time.

Users felt that cylinders were relatively simple to use after proper training; however, the colour-coding for the cylinders from the local supplier was unconventional and confusing. Users felt that the concentrators were straightforward to use after training and simpler to use than cylinders. Challenges included the time required to clean the filter (which they struggled to do regularly), sometimes turning on the wrong flow valve (for models with two outlets), distinguishing small increments on the flow meter and finding concentrators plugged into the mains socket instead of the dedicated UPS socket.

Users estimated that at any given time there are on average about three children and two adults on oxygen (five patients in total, between all three wards). Since the new system was introduced, at least one concentrator has always been in use. A concentrator may be supplying oxygen to a patient for days or even weeks at a time. Overall, users felt the advantages of the new system were better safety (avoiding the hazardous moving of large cylinders), lower cost, logistical convenience (the oxygen source easily moved to the patient instead of having to move the patient to the oxygen source), easier overall use and greater confidence that supplies will never run out. Disadvantages of the UPS concentrator system have been that it cannot be used for patients being transported, and it relies on electricity, which has its own associated costs.

### Performance, maintenance and cost analysis

As of August 2013, the median age of the concentrators was 6.1 years. Of the 14 concentrators acquired since 2004, 12 (86%) were still in service. One was retired after 2476 days in service (approximately 7 years), and one went missing after 2145 days in service. The median duration of operation was 10 702 h, and the combined accumulated duration of operation was 139 538 h. The concentrators underwent 3.5 ± 0.6 preventive maintenance checks per year, with 5% of checks requiring corrective action. The total cost of concentrator maintenance (34 repairs plus regular preventive maintenance) was estimated at US$1361 (US$1067 in parts and the rest in estimated labour time). A total of 32 parts had been replaced since 2006—usually filters and faulty valves, and very rarely sieve beds, compressors or circuit boards. Concentrator downtime due to maintenance was 3.9% of total days in service, but there was no interruption in supply, as functioning units were always available to replace those under repair.

In the Table, the total capital and operating costs of the concentrator system, which has produced approximately 16.7 million litres of oxygen, are compared to the costs of providing the same amount of oxygen with cylinders. For this comparison, we assumed an average output of 2 l/min, which likely underestimates total output considering concentrators can deliver up to 8 l/min and can serve more than one patient simultaneously. Nevertheless, we estimate that by transitioning to concentrators, the MRC has produced oxygen at 49% of the cost of cylinders, based on fully written-off capital costs (i.e., the cost per 1000 l will continue to decrease as long as the concentrators remain in operation), and assuming 10% leakage of cylinder oxygen. Considering the high leakage previously identified in the cylinder system (70%) and typical of piped systems observed elsewhere,^11^ the new system provides oxygen at a conservative estimate of closer to 32% of the cost of cylinders. In the absence of data on UPS maintenance, if we factor in a conservative estimate of 20% of the UPS cost (US$1600), our estimated system cost would increase by 3.5%. We therefore estimate that the MRC has saved over US$45 000 to date by switching to a concentrator-based system.

**Table:**
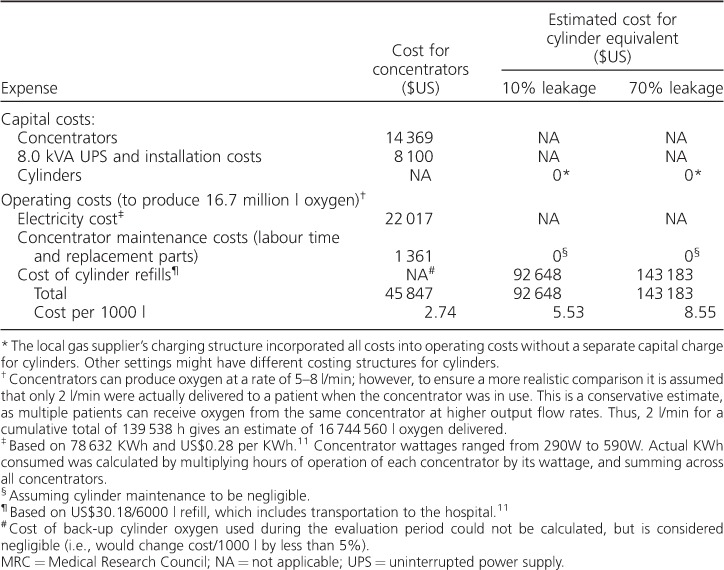
Summary of concentrator expenses at the MRC Hospital, Banjul, The Gambia, and estimated expenses for an equivalent cylinder supply over a period of 8 years

## DISCUSSION

For MRC clinical staff, the most important features of an oxygen system were having oxygen available when needed, safety with minimal hazards, effective use with appropriate training, and it being easy for staff to gain a basic understanding of the equipment.

Oxygen concentrators have been an economical, reliable, and user-friendly source of oxygen at the MRC's hospital since their phased introduction in 2004, having produced an estimated 17 million litres of oxygen over 8 years for less than half the cost of cylinders. The original UPS and most concentrators are still in service after 8 years of operation. Maintenance and repair costs for concentrators have been low, and periodic maintenance by BMETs have had a positive impact on concentrator functionality.

A unique element of this setup is the UPS and dedicated sockets for concentrator use, an appropriate solution for settings where power interruptions are frequent but short in duration (approximately 25% of facilities in The Gambia). In addition to power continuity, the consistency of voltage provided by the UPS will help prolong the life of the concentrators. For settings with longer interruptions, larger capacity battery back-up or solar power are likely to be appropriate.^8^ We have compared the features and costs of such systems elsewhere.^15^,^16^ Back-up cylinders were rarely used.

The other unique characteristic of this evaluation is that we had well-documented and effective support from BMETs who were trained in concentrator maintenance. Such data records are rare in resource-limited settings. More in-depth analyses of oxygen concentrator maintenance at the MRC, including details about the capacity of the maintenance team, the availability of replacement parts and the skills/training required to repair concentrators have been reported elsewhere.^14^,^17^ The scalability of our model would depend on adequate resources allocated to training and equipment maintenance support in routine care settings. Nevertheless, training and maintenance are also required to ensure that the cylinders work properly. Some of the cost savings achieved through the use of concentrators over cylinders (in our case, over US$45 000 over 8 years) could be re-invested in training and building maintenance capacity within hospitals.

We have learned that to maximise continuous function and longevity, periodic user training is needed to ensure that concentrators are plugged into the UPS sockets and that user maintenance (i.e., washing filters) is done. Even when taking into account the added costs of the UPS and long-term concentrator maintenance, oxygen concentrators have offered significant cost savings and an improved user experience compared to cylinders in our setting. Settings where cylinder transport costs are high (e.g., US$11 per refill elsewhere in The Gambia^11^) might expect even greater cost savings by switching to concentrators.

A limitation of this evaluation is that the study was limited to one hospital; however, the analysis period is longer than any reported study of oxygen concentrators in a resource-poor setting. Early concentrator field trials in Mongolia and Egypt,^18^ and more recent national oxygen programmes in Malawi^19^,^20^ and Papua New Guinea,^21^,^22^ have reported positive results on the use of concentrators, but only up to 28 months post-installation. Evidence of the functioning of concentrators beyond 3 years in a low-resource setting is scarce.^14^,^23^,^24^ An additional limitation is the duration of time between the focus group and the recalled events dating back to when cylinders were the primary source of oxygen at the hospital. Finally, our evaluation approach was limited to a comparison of costs to deliver oxygen to patients and did not include patient-level outcomes. This is in keeping with the aims of the evaluation, which did not seek to demonstrate the effectiveness of oxygen. Further studies demonstrating improved patient outcomes will add to the impetus behind improving oxygen availability.

## CONCLUSION

Oxygen concentrators offer significant cost savings and an improved user experience compared to cylinders, even after 8 years of operation. Long-term follow-up data from concentrator-based oxygen supply systems are scarce. Despite being limited to one hospital, the data reported here add considerably to the body of knowledge on the use of concentrators in resource-poor settings. In settings with periodic but short power interruptions, for example where a back-up generator supplements erratic grid supply, a UPS system dedicated to oxygen concentrators will prevent the stop-start operation known to be detrimental to oxygen concentrators, and will ensure a highly reliable supply of oxygen. Some technical support and maintenance, as well as periodic user training, are needed to upkeep the system. This approach can be a model for health systems in settings with similar infrastructure. Long-term follow-up data from larger scale implementation will help to confirm this.
